# Whose voice is heard? Mental health professionals’ involvement, epistemic injustice, and the ethics of psychiatric advance directives

**DOI:** 10.3389/fpsyt.2026.1772856

**Published:** 2026-03-18

**Authors:** Yvonne Quenum

**Affiliations:** Centre Hospitalier Universitaire de Saint-Etienne Pole Psychiatrie, Saint-Étienne, France

**Keywords:** autonomy, epistemic injustice, joint crisis plan, psychiatric advance directive, psychiatric advance statement, physician–patient relations, intersectionality, women’s mental health

## Abstract

Women living with mental health conditions, neurodevelopmental disorders, or other forms of psychosocial disabilities are disproportionately exposed to coercive practices, structural stigma, and epistemic marginalization. Psychiatric advance directives (PADs) have been proposed as tools to promote autonomy and reduce coercion, yet their implementation in practice remains complex. When a mental health professional is designated to support the person in drafting the directive, they may face specific ethical challenges, including the risk of exerting undue influence on the person’s choices. This mini review offers a critical synthesis of theoretical and ethical frameworks to clarify mental health professionals’ roles in facilitating PADs. Drawing on the four models of the clinician–patient relationship developed by Emanuel and Emanuel, and Fricker’s concept of epistemic injustice, we analyze how relational dynamics influence the drafting process. These models provide a useful framework to conceptualize the mental health professional’s role within an alliance aimed at support in restoring agency, trust, and recognition. This analysis highlights how ethically sensitive facilitation may transform PADs into spaces of epistemic repair, ensuring that mental health professionals’ involvement respects women’s autonomy and lived experiences instead of inadvertently reproducing existing power imbalances. This synthesis contributes to socio-political and clinical debates on PADs, care ethics, and health equity, emphasizing the need to recognize and value experiential knowledge. It also underscores the importance of training and having institutional frameworks that empower mental health professionals, particularly nurses, to act as agents of ethical and epistemic justice in supporting the drafting of PADs.

## Introduction

Autonomy has become a central value in healthcare, moving away from paternalistic models and leaning toward greater consideration of patients’ values and choices (1).

While advance directives are well established in end-of-life care, psychiatry remains marked by coercive practices, despite recovery-oriented paradigms and the experiential knowledge of service users (2, 3).

Women living with mental health conditions, neurodevelopmental disorders, or other forms of psychosocial disabilities are particularly vulnerable to coercion and epistemic injustice due to prior exposure to structural violence (4) and persistent difficulties in having their voices recognized (5, 6), often leading to care that is inadequate or unsuitable for their situation (7–9). Psychiatric advance directives (PADs) have therefore been described as promising tools for promoting autonomy and justice in mental health care, particularly among populations exposed to systemic discrimination (10, 11).

However, the implementation of PADs remains uneven. Resources to support PAD completion remain insufficient (12), despite service users frequently expressing the need for assistance in producing them (13). Peer support appears to be a promising strategy for facilitating PADs (14, 15), although its availability varies across contexts and depends on favorable organizational conditions (16). Emerging alternatives such as digital tools or artificial intelligence may improve accessibility but also raise technical, ethical, and equity concerns (17, 18).

Mental health professionals play a crucial role in supporting the drafting, interpretation, and implementation of PADs (19, 20). However, this facilitation occurs within persistent epistemic asymmetries that may shape how patients’ preferences are expressed, interpreted, and enacted. Although PADs are widely presented as tools for promoting autonomy, limited attention has been paid to how clinician-mediated drafting may influence patients’ epistemic agency, including both its empowering and constraining effects. Empirical research remains scarce in this regard, particularly concerning how gendered and intersectional forms of epistemic injustice shape PAD facilitation. These issues are especially critical for women, whose credibility has historically been contested within psychiatric care (8). Addressing this gap requires a conceptual and normative analysis integrating ethical theory, feminist epistemology, and clinical practice.

Drawing on relational models of care (21) and theories of epistemic injustice (22), this Mini Review offers a theory-informed conceptual and ethical analysis of how mental health services and professionals, particularly psychiatric nurses, may shape the epistemic function of PADs when facilitating their drafting with women. The populations concerned are heterogeneous, with distinct clinical trajectories; their grouping reflects a shared exposure to epistemic marginalization due to intersecting structural inequalities related to gender, race, culture, and disability. This category includes cisgender, transgender, and gender-diverse women, as well as those from racialized and culturally diverse backgrounds, whose interactions with psychiatric systems are shaped by these intersecting social identities. This review first examines how epistemic injustice affects women within psychiatric care, then analyzes how professional facilitation of PADs may either support or undermine their epistemic agency and finally discusses the ethical implications and conditions necessary to preserve PADs’ epistemic and rights-based function.

## Methods

This Mini Review adopts a theory-driven narrative and conceptual approach to examine the ethical and relational dimensions of PADs, with a particular focus on epistemic injustice and women’s mental health. Literature searches were conducted in PubMed, Google Scholar, and Cairn between September 2025 and January 2026, focusing on publications from the past 25 years and including English- and French-language articles.

The initial search strategy combined keywords including (“psychiatric advance directive” OR “psychiatric advance statement” OR “joint crisis plan”) AND (“autonomy” OR “epistemic injustice” OR “women” OR “physician–patient relations”). Given the limited number of publications addressing these dimensions simultaneously, additional thematic searches were conducted iteratively for each core concept, including PAD implementation, epistemic injustice, relational models of care, and women’s experiences in mental health systems. Reference lists of selected articles were also examined to identify additional relevant publications.

Publications were included based on their conceptual and analytical relevance to PAD implementation, ethical and relational dimensions of psychiatric care, and epistemic injustice affecting women. Both empirical studies and theoretical or ethical analyses were considered when they contributed to clarifying conceptual relationships, ethical tensions, or implementation challenges.

Rather than aiming for systematic exhaustiveness or quantitative aggregation of findings, this review uses an interpretive synthesis to examine how existing empirical and theoretical literature informs the ethical and epistemic implications of PAD facilitation. This approach was chosen because questions related to epistemic injustice, relational ethics, and autonomy require conceptual analysis and theoretical integration alongside empirical evidence. The objective was therefore to provide a critical and integrative analysis of the literature, rather than a comprehensive systematic review.

## Psychiatric advance directives: definitions

Psychiatric advance directives stem from the idea of a “psychiatric will” proposed by Thomas Szasz (20), further developed in the 1990s and promoted by user associations in the context of the Patient Self-Determination Act (23). The terminology remains non-consensual. In her seminal article on the typology of these documents, Henderson (24) uses the term “advance statement” to describe any document that anticipates preferences in the event of a mental health crisis or incapacity to consent, reserving “psychiatric advance directives” for those with legal force.

Although Henderson’s typology has been widely followed (25, 26), subsequent publications and prevailing usage have extended the term PADs to diverse legislative and organizational contexts (20, 27), including documents with no formal legal status (15). Moreover, while some authors maintain a distinction between PADs and Joint Crisis Plans (13, 28), others consider the Joint Crisis Plans (JCP) to be a form of PAD (18, 29–31), particularly in contexts where the different models are implemented under the same legislative framework. A recent international review further emphasizes that the term PAD is used across markedly different legal frameworks (32).

In the present article, we adopt the definition proposed in this publication, considering PADs as all documents used to declare preferences for treatment in the event of future loss of decision-making capacity, specifically for care related to mental health conditions (32). The preferences expressed can be prescriptive, outlining desired treatments, as well as proscriptive, identifying treatments to be avoided, and the document may also include the designation of a health care proxy (20).

PADs encompass multiple models that vary across legal contexts and in the degree of professional involvement in their drafting (24, 32, 33). Collaborative models, such as facilitated PADs and JCPs, involve clinicians, peer workers, advocates, or relatives in supporting the drafting process through dialogue with users (15, 34–36). Originally designed to include a neutral third party to balance perspectives (37), the JCP is now often developed directly between the patient and a clinician, typically a nurse (29, 38), placing greater ethical responsibility on those professionals to ensure that the user’s preferences are genuinely expressed and respected.

However, publications describing facilitated PADs and JCPs, seldom detail the extent of the facilitator’s influence or clarify who retains the ultimate decision-making authority during the discussion. This ambiguity and the legal context directly affect the articulation between epistemic authority, relating to the recognition of the user’s experiential knowledge, and deontic authority, concerning the power to validate or override expressed preferences (**Table 1**). It may also shape the substance of the document, its underlying objectives, and the way it effectively supports autonomy, particularly for vulnerable populations such as women with mental health conditions.

**Table 1:** Legal framework, conditions of override, and authority implications of psychiatric advance directives (PADs).

Jurisdiction	PAD: Legal framework & enforceability	Conditions of override	Facilitation & professional involvement	Authority implications (epistemic vs deontic)
United Kingdom (England & Wales)	Advance Decisions under the Mental Capacity Act 2005 are legally binding if valid and applicable. Interaction with the Mental Health Act 1983 limits enforceability in certain detention contexts.	May be overridden under the Mental Health Act in compulsory treatment contexts. For ECT, a valid and applicable advance refusal must be respected under MHA s58A.	PAD drafting may occur independently or with clinical support; facilitation practices vary.	PADs may carry deontic authority under the MCA framework, but compulsory mental health powers can reallocate deontic authority to clinicians.
France	No specific statutory framework for PADs; used as clinical instruments within general health law. However, the health care proxy (personne de confiance) is legally recognized under the French Public Health Code and must be consulted when the patient lacks decision-making capacity.	Clinicians may depart from PAD content under compulsory psychiatric care regimes.	PADs may be facilitated by clinicians, relatives or peer workers	Deontic authority remains institutional, while the health care proxy holds a legally recognized role in decision-making processes.
Switzerland	PADs legally recognized under Swiss Civil Code and must be taken into account during involuntary hospitalization when decision-making capacity is lacking.	May be set aside in cases of validity/applicability concerns or where statutory exceptions apply.	PAD drafting may involve clinicians; facilitation models vary across cantons and services.	PADs have recognized deontic weight, though not absolute; authority depends on interpretation and applicability.
Germany	PADs fall under the general advance directive (Patientenverfügung) framework and are legally binding when valid and applicable.	Override primarily limited to questions of validity, applicability, or emergency/compulsory law interfaces.	PADs may be drafted independently; clinician involvement varies in practice.	Strong deontic authority when validity criteria are met; ethical tension shifts to implementation and coercive law interaction.
Australia (Victoria)	Advance Statements recognized under the Mental Health Act; must be considered but are not strictly binding.	Authorized psychiatrist may override if not clinically appropriate or not reasonably practicable. Compulsory ECT requires Tribunal authorization and cannot proceed if the person has capacity and refuses.	Typically clinician-supported; advocacy structures may assist drafting.	Formal recognition of preferences (epistemic), but deontic authority remains primarily with the authorized psychiatrist and Tribunal.
United States (Washington State)	Mental Health Advance Directive statute (RCW 71.32) provides statutory recognition with defined non-compliance conditions.	Providers may decline compliance under enumerated statutory conditions.	PAD drafting may be clinician- or advocate-supported; formal witnessing requirements apply.	Clear statutory allocation of deontic authority with explicit exceptions.
United States (Virginia)	Advance directives recognized under the Health Care Decisions Act; applicability may be limited in detention contexts.	Override possible in emergency custody, temporary detention, or involuntary admission contexts; court authorization required for certain treatments such as ECT in incapacity contexts.	PADs may be completed independently or with professional assistance.	Deontic authority structured by statute but curtailed under compulsory treatment powers.
India	Advance Directives recognized under the Mental Healthcare Act 2017; procedural registration and review mechanisms established.	Subject to review/modification procedures; emergency treatment carve-outs apply. Unmodified ECT is prohibited under the Act.	Clinicians and nominated representatives are involved in statutory processes.	Deontic authority mediated through formal review bodies; effectiveness depends on institutional implementation.

This table summarizes the statutory recognition, conditions of override, facilitation practices, and implications for epistemic versus deontic authority of PADs across selected jurisdictions. It is informed by international comparative work (32) and complemented with data from clinical and legal literature (15, 29).

## Epistemic injustice in mental health care

Epistemic injustice, as conceptualized by Fricker (22), refers to a situation in which people are devalued as subjects of knowledge. It affects people’s ability to be recognized as bearers of knowledge, whether theoretical or experiential (39). She distinguishes between two main forms.

Testimonial injustice refers to the unjustified loss of credibility of an individual due to prejudices related to their identity or social position. Hermeneutic injustice manifests itself when a person lacks the shared conceptual resources that enable them to express or understand their experience, due to prolonged exclusion from knowledge-producing communities. 

A growing body of literature has examined epistemic injustice in mental health care (40, 41), where power asymmetries, the dominance of the biomedical model, and stigma converge to undermine patients’ credibility and partially exclude them from the epistemic space of care (42, 43). Their testimonies may be filtered through assumptions of pathology or “lack of insight” (44). For women with psychiatric disorders, this credibility deficit is often exacerbated by gendered stereotypes portraying them as overly emotional, exaggerating, or less informed (22, 45). Additionally, for women on the autism spectrum, communication differences may lead their speech to be perceived as incoherent or inauthentic, further reinforcing testimonial injustice (46). Such disqualifications may contribute to delayed diagnosis, misinterpretation of distress, and increased exposure to restrictive or inadequate care (7, 43, 47).

Hermeneutic injustice also arises when psychiatric frameworks fail to account for intersectionality—the interplay of gender, ethnicity, disability, and other identities shaping women’s experiences (48, 49). They find themselves deprived of the conceptual resources necessary to make sense of their experiences in accordance with their personal, relational and cultural context (47). Experiences related to coercion, treatment effects, or the reactivation of past traumas, particularly gender-based violence, often remain unrecognized because of their historical invisibility within medical knowledge production.

Recent literature has highlighted specific extensions of epistemic injustice in the psychiatric context. Testimonial smothering refers to the self-censorship adopted by patients who anticipate that their words will be disqualified or misunderstood, and prefer to remain silent rather than expose their experience to further invalidation (41, 50).

Participatory injustice occurs when people are prevented from contributing fully to deliberations or decisions that affect them, not by explicit prohibition, but because of professional norms and institutional hierarchies that limit their effective participation (41, 51).

Institutional epistemic injustice refers to structures that determine which forms of knowledge are recognized in psychiatric care, privileging professional authority while marginalizing experiential expertise (52).

These mechanisms, which particularly affect women, are exemplified by diagnostic overshadowing, whereby diverse complaints are reduced to the lens of an existing diagnosis (44, 53).

Patients’ first-person accounts are then pathologized and stripped of cognitive value, a process reinforced by structural power asymmetries, coercion, and objectivity-driven institutional norms that could marginalize women’s subjective experiences (41, 54).

The consequences are profound and wide ranging. The first is a decline in trust: the credibility deficit leads to disengagement, under-reporting of symptoms and a weakening of the therapeutic alliance. As patients’ voices lose value, the care relationship becomes increasingly asymmetrical and distant, making any collaborative approach difficult and increasing the risk of clinical and diagnostic errors (8, 55).

These dynamics cause moral and social harm: the status of the knowledgeable subject is denied, autonomy is undermined, and the recovery process is compromised. Being continually perceived as an unreliable witness leads to a feeling of loss of dignity, exacerbated by the specific stigma associated with gender and disability (43, 56).

As a result of the stigma surrounding mental disorders, combined with autism-related assumptions of irrationality or incapacity (4, 40), paternalistic and controlling practices may appear justified. Moreover, psychiatric classifications themselves are historically and culturally situated, shaped by value judgments that often reflect gendered and social norms (57). Recognizing these dynamics requires integrating the perspectives of those most affected, such as women whose voices have long been marginalized.

## Epistemic repair through psychiatric advance directives?

Several authors have conceptualized epistemic repair as a process of rebalancing knowledge relations through recognition of users’ experiential competence. Repair may occur through resistance and speaking out (58), the creation of spaces where marginalized voices are heard (59), or by transforming knowledge relations towards mutual recognition (60). In healthcare, promoting the epistemic value of lived experience has been identified as a key avenue for redress (54).

Within this perspective, and drawing on proposals by Sakakibara (41) and Carel and Kidd (61), PADs may represent a potential lever for epistemic repair. Although empirical evidence specific to women remains limited, PADs could plausibly hold relevance for them. By formalizing patients’ preferences in an official document intended to inform future decision-making, PADs could promote epistemic humility (62), encouraging professionals to reflect on credibility biases and to distinguish informed testimonies from presumed symptoms. For autistic women or women with psychiatric disorders, whose emotional, sensory, or relational experiences are often misinterpreted, such formalization may help restore cognitive value to their narratives. The clinic thus shifts from control to co-investigation, and disagreement becomes an opportunity for shared exploration or explanation of the criteria for attributing credibility (41).

Women with intellectual or learning disabilities also face epistemic injustice (63), as differences in communication or cognitive processing are frequently misinterpreted as lack of insight, leading to diminished credibility and increased reliance on proxy decision-making (43). While PADs cannot by themselves resolve these structural inequalities, adapting them to accessible formats and communication needs, may help create conditions that better support the recognition of their experiential knowledge and epistemic agency.

PADs could also function as voice-preserving tools, enabling expression outside time-pressured clinical encounters and supporting forms of communication that may be written, sensory-based, or reflective rather than immediate.

PADs may support relational autonomy (64), by structuring decision-making processes that are co-constructed with patients, their relatives, and multidisciplinary teams. By clarifying preferences and refusals in advance, they could help counter diagnostic overshadowing (53) and lessen the marginalization of patients’ voices during crises. In doing so, they could reinforce patients’ status as epistemic agents within institutional settings.

Beyond testimonial repair, PADs could support hermeneutic repair (39, 65) by providing a shared language for articulating needs that are often invisible or misinterpreted, such as the sensory and communicational experiences of autistic women and women with psychiatric disorders. When developed collaboratively, particularly with the involvement of peer supporters, they can center first-person narratives and foster the co-construction of knowledge between service users and professionals (14, 23).

PADs may also reduce institutional opacity (61) by clarifying rules and channels of communication, enabling individuals to be heard even in crisis situations.

Further research is needed to confirm these potential benefits for women, but in perinatal context, PADs are already used. They offer women with mental health conditions a means to reclaim control over their care during a period of heightened vulnerability, while contributing to suicide prevention (66).

However, the epistemic effects of PADs depend heavily on the relational and institutional conditions of their implementation. Limited participation by the user or a predominance of the institutional perspective could reproduce the power imbalances and epistemic injustices that PADs are specifically designed to correct.

## The four relational models and their implications for support in drafting PADs

The relational models described by Emanuel and Emanuel (21) provide a useful framework for analyzing professional attitudes when supporting the drafting of PADs.

In the paternalistic model, the professional determines what is best for the patient, prioritizing objective wellbeing over expressed preferences. Although this stance is not intended in PADs facilitation, implicit paternalism may arise when power asymmetries or epistemic biases remain unexamined.

In the informative model, decision-making rests entirely with the user, while the professional’s role is limited to providing factual information. However, this presupposes equal access to interpretive resources and sufficient health literacy, which may not hold in contexts shaped by structural inequalities.

In the interpretative model, the professional helps the patient clarify and prioritize their values. For instance, for a woman who expresses that work is a major value, the clinician could offer her therapeutic adjustments that are compatible with this value, in particular avoiding treatments that would prevent her from getting up in the morning. But it could also mean quickly suggesting a work stoppage if she does not want her symptoms to be visible to her colleagues. The decision is made by the user following a discussion based on her values. In an interpretative model, the challenge will be to help her determine which value is most important to her and see how to make choices that best serve her priorities.

The deliberative model goes further, as professionals may share their own health-related values and attempt to persuade patients through dialogue, without coercion. For example, a woman who, during previous decompensation, had a delusional encounter with her children’s school principal, leading to a report that she experienced as distressing and threatening to her maternal role, may subsequently enter discussions with heightened mistrust. When drafting her PAD, she may wish to state her refusal of hospitalization and of treatment that had previously been effective for her. The mental health professional would seek to understand the reasons behind her refusal and help her understand the underlying issues. The mental health professional would also share their own health-related values and attempt to persuade the patient to consider adopting them.

This relational model may require time for reflection and understanding. Thus, when a patient refuses to sign up for a treatment that they have found to be the most effective in soothing them, but which the mental health professional considers to be a good choice for their health, the mental health professional can take the time to explain, understand and allow time for a final decision to be “deliberated”. The role of the mental health professional could also be to suggest alternatives that support the values of the patient and health related values.

## Limitations of relational models

Emanuel and Emanuel (21), acknowledge limitations in each model. The paternalistic model is considered problematic insofar as it does not allow for a shared decision-making process and is primarily suited for decisions made in emergency situations. The informative model assumes stable and transparent values, whereas philosophical accounts of autonomy emphasize the complexity of second-order desires (67, 68). Yet identifying which desires truly express the person’s will remains ethically contested.

## Discussion

### The risk of mental health professional-driven health values

Although interpretative and deliberative models foreground dialogue, they may nonetheless implicitly privilege professionals’ conceptions of “good health”. In psychiatry, where health is often equated with symptom reduction and risk management, tensions arise when patients prioritize other concerns, such as treatment side effects or social roles. Even the definition of “crisis” requires contextualization (69).

### Convincing patients: the risk of a return to paternalism?

Emanuel and Emanuel (21) acknowledge that interpretative and deliberative approaches may slide into implicit paternalism when persuasion becomes normative pressure or even a form of informal coercion (70). A patient may agree to include an intervention in her PAD “for her own good” without fully endorsing it. This tension can be clarified through the distinction between epistemic authority (expert knowledge) and deontic authority (the individual’s right to decide) (71). A patient who ultimately consents to include a treatment in her PAD, without being convinced of the validity of the decision, effectively cedes her deontic authority to the nurse, who holds epistemic authority.

In situations where personal competence, the source of deontic authority, may be questioned due to a psychiatric disorder, a paternalistic relationship can quickly replace other forms of relational models, especially if the mental health professional’s goal is to persuade the patient and the PAD is drafted within hospital premises. In this context, mental health professionals’ epistemic authority creates an imbalance that fosters epistemic injustice.

This imbalance may be reinforced in precision and genetic psychiatry settings, as these approaches carry strong epistemic power and can exacerbate testimonial shadowing, when women feel that their lived experiences, including those shaped by gendered realities, are not acknowledged or do not align with the expert’s scientific data. It may also complicate meaningful consent (72).

By providing a subjective space to share individual experiences and personal choices as valid knowledge, PADs could be particularly valuable in these contexts, when their facilitation is guided by ethical sensitivity and awareness of relational dynamics.

Epistemic hierarchies also operate within institutions. Psychiatric nurses, most of whom are women (73), may struggle to have patients’ expressed wishes fully recognized within medical decision-making structures (52), thereby invalidating their holistic assessment of the situation and their caring-centered approach (74). This internal imbalance challenges the plurality of perspectives necessary for supporting the recognition of diverse lived experiences.

### An ideal model?

Emanuel and Emanuel (21) identify the deliberative model as the ideal form of the patient–physician relationship. However, this claim warrants reconsideration in light of concerns about epistemic injustice and deontic authority, as well as the evolution of patient participation since 1992. Moreover, these reflections emerged from a seminar setting, and it remains unclear whether service users were included in defining this relational ideal.

### Autonomy: a responsibility and a burden for the most vulnerable?

While PADs promote consent-based care, autonomy may become burdensome for individuals experiencing acute vulnerability, as drafting a PAD requires reflection and deliberation that may be difficult in such circumstances (75).

Survivors of psychiatry have made critique toward certain practices inspired by the recovery philosophy, when they are “co-opted” by psychiatric institutions and neoliberal logic (76–78). This analysis could be especially relevant for women living with mental health conditions, often situated at the intersection of multiple forms of discrimination. They disproportionately experience economic, wage, and housing inequalities, and are more likely to suffer traumatic events, which further increases their vulnerability. It is therefore crucial not to individualize these difficulties or shift responsibility onto these women in the name of promoting autonomy.

### A tool for care, a tool for rights

The facilitator’s stance largely reflects how the PAD is understood: as a therapeutic instrument for crisis prevention and care optimization, or as a rights-based mechanism affirming the individual’s will and preferences. Historically rooted in user movements and emancipatory thought, PADs aim to redistribute decision-making power.

However, their institutionalization within contemporary psychiatry takes place within specific medico-legal and organizational environments that shape their practical meaning. Ethical agency in mental healthcare is not exercised in a vacuum but is conditioned by systemic vulnerability, institutional fragmentation, and professional liability pressures (79). In contexts where clinicians are increasingly held accountable not only for diagnostic and therapeutic choices, but also for patients’ behaviors, defensive practices may emerge as adaptive responses to perceived legal risk (80).

Within such climates, PADs may see their subversive potential diluted. They can gradually be reframed as crisis prevention and risk management devices rather than serving as mechanisms for reconfiguring power asymmetries between mental health professionals and service users. They risk being incorporated into regulatory frameworks focused on hospitalization reduction and organizational logics of control and accountability. This shift does not necessarily reflect ill intent on the part of clinicians, but rather the influence of institutional cultures that prioritize liability management and procedural compliance over relational transformation (79).

This ambiguity regarding the aims of PADs has also been identified as a key obstacle to their implementation, leading, in some contexts, to their reframing as crisis-management tools rather than rights-based instruments (81).

This ethical tension between promoting users’ rights and fulfilling professional obligations of care and beneficence, partly explains the resistance observed among some professionals, who fear that PADs may be used to refuse necessary treatment (82). However, content analyses have shown that service users generally included clear, understandable, and clinically relevant information in their PADs, often explaining the underlying reasons for their preferences. These reasons were related to previous adverse drug reactions and personal experiences of hospitalization. Only a very small minority indicate a refusal of all psychiatric medication, and the overall content of PADs tends to remain consistent with standard clinical practice (83).

Interestingly, a study in which psychiatrists and psychiatric nurses drafted PADs for themselves revealed that 30% refused neuroleptics and 46% electroconvulsive therapy (84). This paradox reveals a shared ambivalence toward specific interventions: while professionals may fear that service users will reject all care, they themselves express preferences and limits when imagining their own vulnerability. Such findings underscore the importance of institutional safeguards capable of protecting the rights-based orientation of PADs from being subsumed under defensive or risk-management imperatives (79).

## Safeguards for epistemic integrity in PADs

While empirical evidence remains limited, several safeguards may help mitigate the risk of reproducing existing epistemic and institutional asymmetries during the drafting and implementation of PADs.

### Normative and legal safeguards

A clear legislative framework protecting patients’ deontic authority may help reduce epistemic and institutional asymmetries in the implementation of PADs. To fulfill their ethical and epistemic function, PADs need to be accessible to the widest possible population, which requires the involvement of all mental health professionals in supporting their drafting. Training for mental health professionals could incorporate patients’ rights, relational ethics, and epistemic injustice, while creating reflective spaces that foster awareness of professional influence, credibility judgments, and relational power dynamics. Peer workers and nursing champions (81) may play a key role in supporting these spaces. Such approaches may help preserve PADs as tools that support autonomy and recognition of experiential knowledge.

### Organizational safeguards

More horizontal institutional models may facilitate a more balanced distribution of epistemic authority. Evaluation frameworks could extend beyond crisis-management indicators to include measures related to autonomy, shifts in power dynamics, and patient-perceived agency. Involving people with lived experience and peer support workers in governance, training, implementation, and evaluation may further strengthen this orientation.

Formal recognition of the role of nurses and other frontline professionals in PAD facilitation, including their participation in multidisciplinary discussions and decision-making processes, may help ensure that patients’ expressed preferences are effectively communicated and considered. Providing protected time and institutional support for PAD facilitation may also reduce the risk that these processes are constrained by organizational or managerial priorities. Clear procedures are needed to ensure that the content of PADs is meaningfully considered during crises, including mechanisms for documenting and reviewing situations in which patients’ expressed preferences are not followed.

### Procedural safeguards in facilitation

Service users should ideally retain control over key aspects of PAD drafting, including timing, facilitation, and the option to complete the document independently. The most important thing is that users can decide with whom they draft their PAD and that they feel confident with that person (81). Professional support may be most ethically appropriate when aligned with the individual’s expectations and conception of autonomy.

In this perspective, nurses and other frontline professionals are not merely technical facilitators but ethical mediators. Such safeguards may be particularly important for populations whose voices are frequently marginalized, including women.

## Limitations

This mini review adopts a narrative and conceptual approach, which entails an inherent risk of selection bias in the identification and interpretation of the literature. The jurisdictional analysis is not exhaustive and highlights the uneven legal coverage across countries. In addition, empirical data remains limited, particularly regarding sex and gender specific analyses of PAD implementation. The normative arguments developed here are inherently value-laden and grounded in an ethical framework centered on epistemic justice and relational autonomy. Finally, this analysis is written from the standpoint of a clinician; incorporating the perspective of service users in the development of this work would have enriched its scope and strengthened its critical depth.

## Conclusion

PADs occupy a unique position at the intersection of care and rights, offering individuals a structured means to document their preferences within systems that have historically marginalized their voices. While empirical evidence specific to women remains limited, PADs may hold particular relevance for women with psychiatric and/or neurodevelopmental conditions, as they could help counter epistemic injustice by legitimizing experiential knowledge within clinical decision-making processes.

However, PAD drafting and implementation are shaped by power asymmetries, professional epistemic authority, and institutional constraints. Peer support can play an important role in preserving narrative integrity and supporting patients’ epistemic agency. In contrast, while nurses’ involvement is essential to ensure accessibility and implementation, it may also reproduce epistemic asymmetries unless supported by training and organizational frameworks explicitly attentive to epistemic injustice. Without nurses training and support, PADs risk remaining underutilized or reduced to formalities disconnected from patients’ needs.

PADs could help mitigate certain forms of epistemic injustice experienced by women, but they cannot, on their own, resolve structural power asymmetries without broader institutional accountability.

Future research should adopt participatory approaches and examine how PADs are implemented in crisis situations. Empirical studies are needed to assess whether the preferences expressed in PADs are respected in practice, and how patients’ deontic authority is upheld or overridden by professional epistemic authority when clinical decisions must be made. Such work is essential to determine whether PADs function as genuine instruments of self-determination or remain constrained by existing institutional power dynamics.

Moreover, it is crucial to integrate an intersectional perspective to recognize and respond to the overlapping experiences of gender, disability, culture, and other forms of vulnerability, thus paving the way for psychiatry that is truly equitable, inclusive, and humane.
